# Oral *Huzhang* granules for the treatment of acute gouty arthritis: protocol for a double-blind, randomized, controlled trial

**DOI:** 10.1186/s13063-022-06188-x

**Published:** 2022-04-01

**Authors:** Mi Zhou, Liang Hua, Yi-Fei Wang, Si-Ting Chen, Chun-mei Yang, Ming Zhang, Xin Li, Bin Li

**Affiliations:** 1 Department of Dermatology, Yueyang Hospital of Integrated Traditional Chinese and Western Medicine, Shanghai University of Traditional Chinese Medicine, Shanghai, 200437 China; 2 Institute of Dermatology, Shanghai Academy of Traditional Chinese Medicine, Shanghai, 201203 China

**Keywords:** Gouty arthritis, Randomized controlled trial, *Huzhang* granules, Traditional Chinese medicine

## Abstract

**Background:**

Acute gouty arthritis (GA) is the main clinical manifestation and the most common initial symptom of gout. The treatment of acute GA involves the use of colchicine, non-steroidal anti-inflammatory drugs, and corticosteroids. Because of the side effects of these drugs, their clinical applications are limited. The use of traditional Chinese medicine for the treatment of acute GA has unique advantages. The aim of this trial is to clarify the treatment efficacy, safety, and recurrence control efficacy of *Huzhang* granules (HZG) in patients with GA showing dampness-heat syndrome.

**Methods/design:**

This double-blind, randomized, controlled trial was planned to be conducted between July 1, 2020, and December 31, 2022. A sample size of 267 participants (89 per group) with GA will be randomly assigned to three treatment groups in the ratio of 1:1:1: HZG, etoricoxib, and placebo groups. The study duration is 13 days, including a 1-day screening period, 5-day intervention period, and 1-week follow-up period. The primary outcome is analgesic effectiveness, assessed as pain in the worst-affected joint, which will be measured using the visual analog scale. Secondary outcomes include the patient’s assessment of pain in the primary study joint, patient’s global assessment of response to therapy, investigator’s global assessment of response to therapy, investigator’s assessment of tenderness and swelling of the study joint, and TCM syndromes. Furthermore, the number, nature, and severity of adverse events will be recorded.

**Discussion:**

This study will provide evidence regarding the clinical efficacy and safety of Chinese medicine treatment for acute gouty arthritis. This study will provide noteworthy findings.

**Trial registration:**

ClinicalTrials.gov NCT04462666. Registered on July 05, 2020 (first version).

## Background

Gout is a recurrent chronic inflammatory disease caused by monosodium urate (MSU) crystals [1]. Acute gouty arthritis (GA) is the most common symptom of gout. The frequency of acute attacks increases with disease progression, and joint destruction may occur [2, 3]. The pooled prevalence of hyperuricemia in China from 2000 to 2014 was approximately 13.3%, and the pooled prevalence of gout was 1.1%; hyperuricemia seriously affects the quality of life, resulting in large economic costs and mental stress [4]. Healing gout is often an unachieved goal [5–7].

Acute GA is the main reason that patients with gout visit a doctor. The main pathological change is acute inflammation of the joint and its surrounding tissues caused by the precipitation of MSU crystals [8]. The treatment of acute GA involves the use of colchicine, non-steroidal anti-inflammatory drugs (NSAIDs), corticosteroids, or a combination of any two agents [9]. For severe refractory GA, anti-tumor necrosis factor-α or anti-interleukin-1β monoclonal antibodies can be used [10, 11]. However, these agents are associated with severe potential adverse effects (AEs) and risk of drug–drug interactions, especially in elderly patients and those with chronic renal insufficiency or diabetes [12]. In addition to these conventional treatments, traditional Chinese medicine (TCM) has attracted considerable attention because of its efficacy and low incidence rates of side effects [13, 14].

The use of TCM for the treatment of GA has been associated with satisfactory therapeutic effects, with fewer AEs and minimal toxicity [15]. In TCM, GA belongs to the Bi pattern category, and the primary cause is the invasion of wind, cold, dampness, heat, or other pathogenic factors. Among these factors, dampness and heat are the most common external causes of GA. The main TCM treatments for GA include clearing heat and removing dampness, as well as promoting blood circulation and removing blood stasis [15]. The modified Simiao decoction has demonstrated effectiveness in the treatment of GA by eliciting anti-inflammatory effects and lowering urate levels [16].

*Huzhang* granules (HZG), a Chinese herbal prescription, are composed of 12 ingredients (*Polygonum cuspidatum*, *Notopterygium*, *Angelicaepubescentis*, *Angelica*, *Ligusticum wallichii*, *Rhizomaatractylodis*, *Cortex phellodendri*, *Bidentate achyranthes*, *Capillary wormwood*, *Stephania tetrandra*, *Tuckahoe*, and *Cynanchumpaniculatum*) (Table 1). The predecessor of HZG is the *Huzhang Tongfeng* decoction, which was formulated in the 1980s by Xia Han (a well-known Chinese surgeon) and has been used in the clinical treatment of gout for over 30 years at the Yueyang Hospital of Integrated Traditional Chinese and Western Medicine affiliated to Shanghai University of TCM. According to the syndrome differentiation approach in TCM, there are four syndromes of GA: wind-dampness-heat, damp-turbidity, blockage of phlegm and blood stasis, and chronic obstruction due to deficiency. These four syndrome types correspond to the four stages of GA: acute stage, remission stage, chronic stage, and later stage. The method of clearing heat, removing dampness, and dredging collaterals is generally used for the treatment of acute GA [14, 16]. HZG was composed on the basis of this method.

**Table 1:** Ingredients of *Huzhang* granules (intervention drug) with English translations

Main composition	Latin scientific name	Plant part	Amount (g)
*Polygonum cuspidatum*	*Reynoutria japonica Houtt.*	Rhizome	15
Notopterygium	*Notopterygiumincisum Ting ex H. T. Chang*	Rhizome	9
Angelicaepubescentis	*Heracleum hemsleyanum Diels*	Rhizome	9
Angelica	*Angelica sinensis (Oliv.) Diels*	Root	12
Ligusticumwallichii	*Ligusticum chuanxiong Hort.*	Rhizome	10
Rhizomaatractylodis	*Atractylodes Lancea (Thunb.) DC.*	Rhizome	12
Cortex phellodendri	*Cortex Phellodendri Chinsis*	Bark	12
Bidentate achyranthes	*Achyranthes bidentata Blume.*	Root	12
Capillary wormwood	*ArtemisiacapillarisThunb.*	Stem and foliage	15
Stephania tetrandra	*Stephania tetrandra S. Moore*	Root	9
Tuckahoe	*Poria*	Sclerotium	15
Cynanchumpaniculatum	*Cynanchumpaniculatum (Bunge) Kitagawa*	Root and rhizome	15

Our data from clinical studies confirm the effectiveness of HZG in the treatment of GA, with 87.5% of the patients showing improvement in joint swelling and pain. Moreover, HZG can significantly reduce white blood count, erythrocyte sedimentation rate, and C-reactive protein and interleukin-6 levels in patients with GA [14]. However, because of the complex composition of HZG, the mechanism underlying its therapeutic action remains unclear.

The beneficial effects of HZG on acute GA are well known and are considered to be derived from the improvement of wind-dampness-heat syndrome, leading to improvement of joint inflammation. However, no large-scale randomized controlled trials have been conducted on the efficacy, safety, and recurrence control efficacy of HZG. Therefore, we aim to conduct a double-blinded, randomized, controlled clinical trial to evaluate the efficacy of oral HZG for the treatment of patients with acute GA showing wind-dampness-heat syndrome.

## Methods

### Design

This study is designed as a three-parallel group, double-blind, randomized, controlled clinical trial. The objective is to clarify the clinical efficacy, safety, and recurrence control efficacy in patients with wind-dampness-heat syndrome. The research findings will be applied to establish a clinical standard for the treatment of gout. At the time of the writing of the trial protocol (version 1.0, April 09, 2020), enrollment for the trial was yet to begin. Recruitment was expected to start on July 1, 2020, and end on December 31, 2022.

We aim to recruit individuals from the Shanghai region, China. Patients with acute gout requiring primary and secondary care, including inpatients who develop an acute gout attack, will be recruited. Recruitment will occur in the secondary care setting with treatment occurring on an outpatient basis, or it can occur in secondary care if symptoms warrant admission or if the participant is already an inpatient and develops an acute gout attack. A total of 267 people will participate in this clinical trial, and each participant can only be enrolled once. The study will be conducted at the Shanghai Yueyang Integrated Medicine Hospital.

The study consists of five phases: screening/enrollment, allocation, treatment/intervention, end of intervention, and follow-up. In the enrollment process, participants will be recruited via a gout specialist clinic for physical examination and eligibility assessment. The time between assessment and intervention should not exceed 1 week (week 0). If more than a week has passed since the assessment, the assessment of the participant will be repeated before the intervention. If eligible, the participants will be requested to sign the written informed consent regarding participation in the trial (procedures, risks, options for dropping out), the use of laboratory data, and collection, storage, and use of biological specimens. Details of informed consent will be explained by the study investigators or medical staff members with adequate training. Once an informed consent form is signed and dated by the participant, a participant identification number will be assigned to facilitate participant identification.

**Fig. 1:**
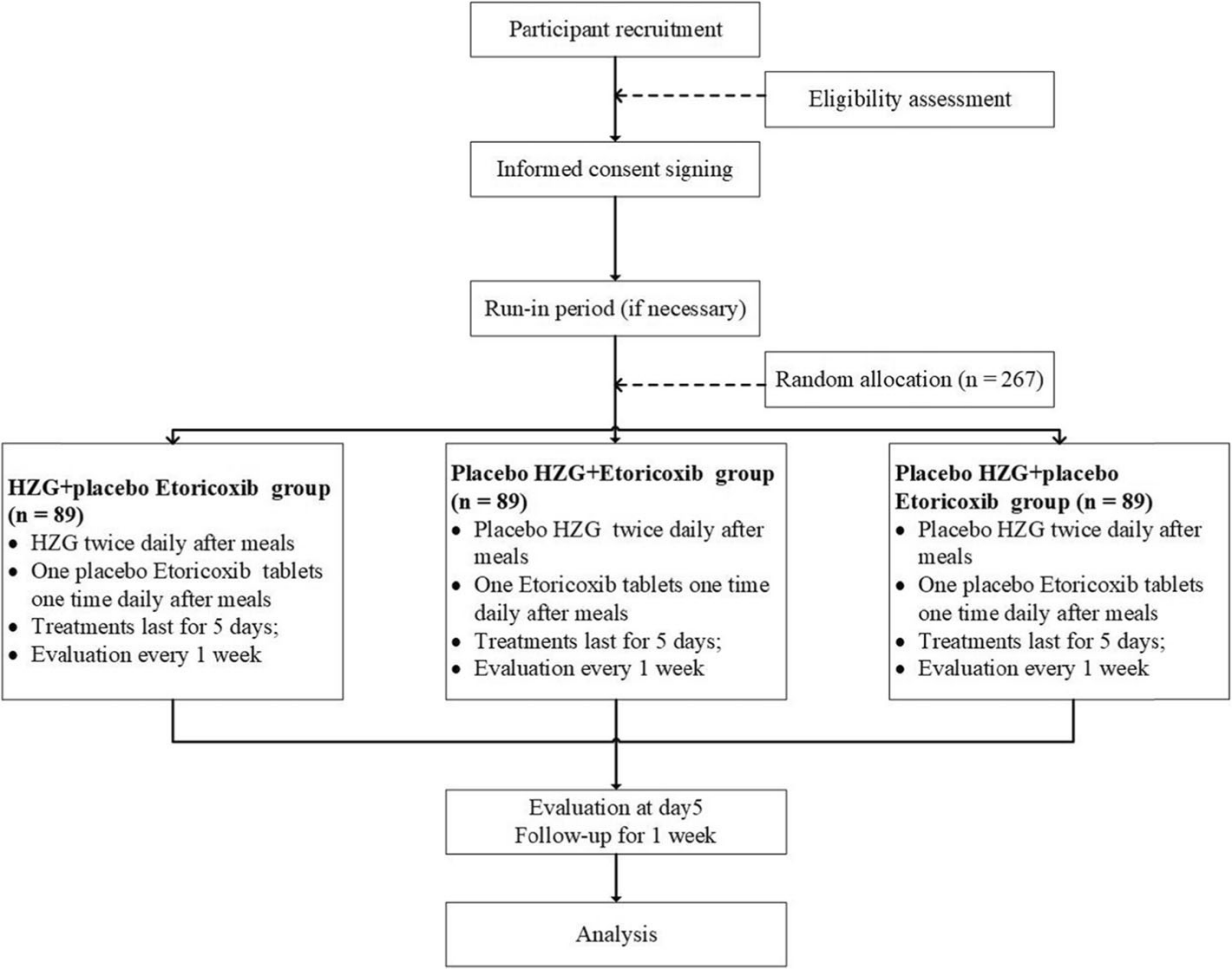
Flow diagram showing progress through the study

Participants will be randomly allocated to HZG, etoricoxib, or placebo groups and undergo corresponding intervention over the course of the 5-day treatment period. We will treat the participants in the HZG group with HZG and etoricoxib placebo tablets, those in the etoricoxib group with etoricoxib tablets and HZG placebo, and those in the placebo group with HZG placebo and etoricoxib placebo tablets (Fig. 1). The participants will receive systemic therapy according to the judgment of their treating physicians. We will record all changes in symptoms, prescriptions, relevant scores, macroscopic characteristics (based on photographic evidence), and any AEs. This placebo-controlled study will verify whether the curative effect of HZG is better than that of a placebo, which will adequately test the efficacy of HZG. This study design applied in such a validation is superior.

The trial protocol was approved by the Shanghai Yueyang Integrated Medicine Hospital ethics committee and registered with the Clinical Trial Registry (NCT04462666).

### Patient and public participation

Participants and the public were not involved in the design or recruitment of this clinical trial. In the preparation phase of the trial, we fully considered the time cost of follow-up for each patient. In the data management system, we added an option to upload patient laboratory test reports via a mobile phone and set up treatment follow-up reminders to improve adherence. At the time of signing the informed consent form, participants will be informed of the burden of the study and their freedom to withdraw from the study at any time. Upon completion of the study, all participants will receive feedback concerning the assessment results.

### Eligibility criteria

Inclusion criteria:

(i) Diagnosis of acute gout arthritis as defined by the American College of Rheumatology 1977 preliminary criteria,
(ii) Male or non-pregnant, non-nursing female individuals,
(iii) 18–70 years of age,
(iv) Occurrence of gout attack ≤ 48 h previously,
(v) In the week before this observation, non-steroidal anti-inflammatory drugs, analgesic, drugs, and drugs affecting uric acid metabolism were not used,
(vi) Subjects capable of providing informed consent,

Exclusion criteria:

(i) Failure to meet diagnostic criteria,
(ii) Evidence of uncontrolled concomitant cardiovascular, neurological, hepatic, or gastrointestinal disease—potential participants who have active concomitant disease can only be eligible after discussion and agreement with the treating medical team,
(iii) Patients in a critical condition that makes it difficult to evaluate the effectiveness and safety of clinical observations,
(iv) Severe deformity, stiffness, and labor loss in patients with advanced arthritis,
(v) Known allergy to drugs used in this study.

### Interventions

#### HZG intervention

Participants in the experimental group will receive 10 sacks of HZG granules. They will be instructed to take two sacks per day, one in the morning and one in the evening, approximately 30 min after the meal. The major ingredients of the HZG are listed in Table 1. Placebo etoricoxib will also be administered daily in the morning for 5 days.

#### Etoricoxib intervention

Participants in the etoricoxib group will receive five etoricoxib capsules. They will be instructed to take one capsule per day in the morning, approximately 30 min after eating. The placebo HZG will also be administered two sacks per day, one in the morning and one in the evening, approximately 30 min after the meal.

#### Placebo intervention

Participants in the placebo group will receive 10 sacks of placebo HZG. They will be instructed to take two sacks per day, one sack in the morning and one in the evening, approximately 30 min after the meal. Placebo etoricoxib will also be administered daily in the morning for 5 days.

All patients in each of the treatment groups will be provided free medications and laboratory tests, as well as transportation subsidies, and we anticipate that this will contribute to enhancing patient compliance. During the follow-up of the treatment period, each participant will be requested to record the use of drugs, and they will be advised to return any unused drugs to the hospital during the last follow-up visit.

### Outcome measures

Through the implementation of deterministic, double-blind, double-simulated randomized controlled trials, we can provide clear guidance for the safe management of patients with acute gout. Information from the proposed outcome metrics will be collected for further studies with larger study populations. Recruitment and retention rates, the proportion of patients who do not meet the criteria, and the willingness of patients to be randomly assigned will be calculated. Compliance and compliance rates and qualitative feedback will be checked. Economic data on the use of medical resources and health-related quality of life will be collected and analyzed. Safety outcome measures will be reported in accordance with the requirements of a clinical trial designed to study a drug regimen.

#### Primary outcome

The primary outcome is analgesic effectiveness, assessed as pain in the worst affected joint, which will be measured using the visual analog scale at rest and with activity. The patients will be evaluated from baseline to 5 days post-randomization (4 h, day 1, day 2, day 3, day 4, and day 5) and during the follow-up period. The primary efficacy endpoint will be the assessment of joint pain on treatment days 2–5.

#### Secondary outcome

The proposed secondary outcome measures consist of Likert scales for the assessment of joint tenderness and swelling. The Likert scale scores of the patients will be assessed during the treatment period (4 h, day 1, day 2, day 3, day 4, and day 5) and the follow-up period. Symptom relief time, patient satisfaction, and 36-item Short Form Survey (SF-36) data will be collected at baseline and on day 5. C-reactive protein and inflammatory cytokine (IL-1, IL-6, IL-8) levels, vital signs, routine blood tests, routine urine tests, electrocardiograms, and blood biochemical parameters will be assessed at baseline and on day 5.

During the treatment period, combined medication, AEs (including dizziness, drowsiness, nausea, vomiting, abdominal pain, indigestion, rash, xerostomia, and any other symptoms reported by the patient), and serious adverse events (referring to adverse events requiring hospitalization) will be recorded. Patients may be withdrawn or returned to the standard of care at any time if there is a significant clinical indication to do so or at the participant’s request. Throughout the intraoperative and immediate postoperative period, the study team will communicate with the clinical team to ensure protocol adherence and safety.

### Sample size

The required sample size was calculated using an estimation formula based on the differences among the three sample rates [14, 17, 18]. A previous clinical study [19] reported that the response rates of the etoricoxib treatment, TCM treatment group, and placebo treatment groups were 63.89%, 42.5%, and 15.5%, respectively. Setting the two-sided significance level (*α*) at 0.05 and statistical power at 0.8, a minimum sample size of 74 participants per group (222 participants in total) was estimated to provide sufficient statistical power to detect a between-group difference of approximately 20% in treatment efficiency, defined as the change in analgesic effectiveness. Considering a 20% loss to follow-up, we aim to enroll 89 patients in each arm, that is, a total of 267 patients.

### Randomization and allocation

Once eligibility is confirmed and consent is obtained, the participants will be randomized into the study arms/groups using non-stratified permuted blocks of varying lengths (*n* = 6 blocks; patient distribution = 1:1:1). Randomization will be conducted using an internet-based randomization system. Once a patient has consented to participate in the trial, the designated staff will log in into the randomization system web page to confirm eligibility; a random allocation will be sent to the pharmacy.

A responsible unit unrelated to this clinical trial will be designated to complete the packaging and distribution of the drugs (test drugs and control drugs) under the supervision of the statistician. Allocation concealment will be ensured. The randomization codes will be distributed through a data network platform designed by the Data Management Center of Jiangsu Famaisheng Medical Technology. Afterwards, participants will be randomly assigned to different treatment groups.

### Treatment cycles

The treatment is planned for 5 days in one cycle. All interventions will be stopped after the 5-day treatment period and 1-week of follow-up.

The pain associated with acute GA is self-limiting; that is, it can resolve on its own after approximately 1 week without medication. The participants will be informed on the consent form that if they cannot bear the pain, they can withdraw from the study at any time and choose other treatment options.

### Test drugs and blinding

The study drug and its placebo are provided by China Resources Sanjiu Medical & Pharmaceutical Co., Ltd. Etoricoxib will be purchased from Merck and Co., Inc. The placebo of etoricoxib was manufactured by Nanjing Hengzheng Pharmaceutical Research Institute Co., Ltd., which is responsible for all processes of placebo production and production processing from ingredients to aluminum molding.

All trial investigators will be blinded to the interventions. Pharmacy staff will maintain a record of the treatments administered. All trial participants, care providers, and outcome assessors will be blinded to treatment. Each treatment has an equivalent placebo to ensure that blinding is maintained throughout the study.

All investigators involved in the trial will be blinded to treatment. Patients will not be informed of their assigned treatment during the study. Pharmacy staff will keep a record of allocated treatment arms in the event of emergency unblinding.

The treatment code for a participant can be broken by any clinician, either directly or via contact with the principal investigator. Allocation lists will be made available to the site that will be provided 24-h cover by the pharmacy. Thus, the central pharmacy can access the allocation list. Where possible, the local investigator should aim to discuss the need for unblinding with the coordinating investigator, and attempts to preserve blinding of relevant research staff (data collection, analysis, and interpretation) will be made. The coordinating investigator is responsible for pharmacovigilance management and reporting.

### Measurements

Demographic data such as age, sex, ethnicity, race, and body mass index will be recorded. Individuals will receive the following instructions during the trial period: no alcohol, low-purine, low-fat diet, and plenty of water (≥ 2000 ml/day); avoid predisposing factors such as joint cold, trauma, and excessive fatigue; and avoid use of diuretics, salicylic acid preparations, glucocorticoids, and other drugs that affect uric acid metabolism and excretion. Before the beginning of treatment and after 5 days of treatment, medical histories will be recorded for each patient, and laboratory examinations will be performed and will include the following: C-reactive protein, interleukin, blood routine, blood biochemistry, routine urine test, and electrocardiogram. In addition, the tenderness, redness, and swelling of the involved joint, symptom relief time, patient satisfaction, and 36-Item Short Form Survey values will also be collected. The study team will build and maintain electronic case report form data and ensure data completeness and quality periodically by means of internal audits. Double data entry will be used to ensure data quality. All efforts will be made to maintain confidentiality of patient data by using multiple means, such as de-identification, use of password-protected secure servers, and restriction of access to study team members.

### Drug combination

In this study, all drugs will be considered as combined drugs. Details including trade name, dosage, indications, and duration of medication will be recorded in the case report form. Whether a participant should withdraw from the trial because of the nature of the combined drugs will be judged by the study investigators. The use of diuretics, salicylic acid preparations, glucocorticoids, and other drugs that affect uric acid metabolism and excretion will be avoided.

### Statistical analysis

The statistical analysis plan was developed by professional statisticians upon consultation with the main trial investigators. The data will be stored at the data management center of Jiangsu Famaisheng Medical Technology Co., Ltd., and processed by in-house statisticians blinded to group allocation. The analyses will be conducted using SAS version 9.2 (SAS Institute Inc., Cary, NC, USA) and will cover the number of participants enrolled in each group, the number of patients who dropped out of the study and the reason for the dropout, demographic and other baseline characteristics, compliance, efficacy analysis, and safety analysis.

Descriptive and comparative analyses will be performed. Qualitative data will be described using frequency tables, percentages, or constituent ratios and compared using the chi-squared test, Fisher’s exact probability test, Wilcoxon rank-sum test, Cochran–Mantel–Haenszel chi-square test, and weighted least squares covariance. Quantitative data will be described using the mean, standard deviation, median, quartile thresholds, and minimum and maximum values of the range. The *t*-test will be used for comparative analysis of data showing normal distribution; it will be used with Satterthwaite correction if the variance is uneven. Quantitative data exhibiting non-normal distribution on the Wilcoxon rank-sum test will be analyzed using the Wilcoxon signed-rank sum test and covariance generalized linear models. The main analyses include an intention-to-treat (ITT) analysis and a per-protocol subject analysis of the primary outcomes. The ITT analyses will include all randomized patients. The last observation carried forward method will be used in the ITT analysis for missing data imputation. Subgroup analyses will be performed based on the severity of the disease. All hypothesis tests will be two-sided. The test statistics and corresponding *P*-values will be reported. Statistical significance will be established at *P*< 0.05, with a high statistical significance established at *P*< 0.01.

### Adverse events

The Chinese herbal medicines contained in HZG are listed in the Pharmacopeia of the People’s Republic of China. The dose proposed for use in this study is within the range recommended by the Pharmacopeia Commission of the Ministry of Health of the People’s Republic of China. To date, no adverse reactions have been reported in relation to the clinical application of HZG.

Blood and urine samples, liver function, renal function, and electrocardiograms will be examined before the start of treatment and after 5 days of treatment. Researchers will focus on identifying any abnormal changes in the results. All AEs will be collected and graded by the assessor at each visit to determine the severity and potential relationship to treatment. The safety assessment includes the incidence of treatment-induced or serious adverse events, withdrawals due to adverse events, and changes in laboratory parameters. All drugs in this trial will be immediately discontinued in the event of a serious adverse event. The specific implementation of such measures is presented in Table 2. In addition, all severe AEs will be reported to the first responsible staff member and to the ethics committee within 24 h.

**Table 2:** Schedule for enrollment, intervention, and assessment

	Phase	Screening/enrolment	Allocation	Treatment/intervention					End of intervention	Follow-up
Activity	Time points	Day 0		Day 1	Day 2	Day 3	Day 4	Day 5		Week 1
**Screening/enrolment**	**Eligibility screening**	X	X							
	**Obtaining informed consent**	X								
	**Clinicopathological evaluation**	X								
	**Medical history taking**	X								
	**Enrolment**	X								
	**Random allocation**		X							
	**Biological specimen collection**		X						X	X
**Treatment/intervention**	**HZG+ placebo etoricoxib**			**★----------------------------------------------------★**						
	**Etoricoxib + placebo granules**			**☆----------------------------------------------------☆**						
	**Placebo granules + placebo Etoricoxib**			**○----------------------------------------------------○**						
**Outcome assessment**	**VAS score**		X					X	X	X
	**Tenderness, redness, and swelling of the involved joint**		X					X	X	X
	**Symptom relief time**		X					X	X	X
	**Patient satisfaction**		X					X	X	X
	**36-Item Short Form Survey**		X					X	X	X
	**CRP**		X					X	X	
	**IL-1**		X					X	X	
	**IL-6**		X					X	X	
	**IL-8**		X					X	X	
**Safety assessment**	**Vital signs**		X					X	X	
	**BR**		X					X	X	
	**BB**		X					X	X	
	**RUT**		X					X	X	
	**DC**		X					X	X	
	**ECG**		X					X		
	**PE**		X					X	X	
	**AEs**		X	X	X	X	X	X	X	
	**Severe AEs**		X	X	X	X	X	X	X	

*✩****○*** intervention in the control group, *★* intervention in the experimental group, *HZG Huzhang* granules, *VAS* Visual analog scale, *CRP* C-reactive protein, *IL* Interleukin, *BR* Blood routine, *BB* Blood biochemistry, *RUT* Routine urine test, *DC* Drug combination, *ECG* Electrocardiogram, *PE* Physical examination, *AE* Adverse event

### Termination and withdrawal

The trial will be terminated for any participant who develops one or two of the following conditions during the trial: (1) intolerable side effects and (2) serious acute or chronic organic disease. Any participant can withdraw from the trial at any time and for any reason without affecting the current or future treatment. Investigators will attempt to contact any participant who withdraws or discontinues to complete the final evaluation. The reasons for withdrawal or termination and the time of the last dose will be recorded. All withdrawals and terminations will be reported and analyzed.

### Data management and monitoring

All physicians, assessors, and research assistants will attend training workshops before the trial. Data entry will be completed using the case management system specifically designed for this trial by Jiangsu Famaisheng Medical Technology. Data collection will include all the information in the case report forms. The data will be entered using the double-entry method. To ensure the quality and consistency of the source data and of the data entered into the database, two researchers will independently check the source data and compare them against the information entered into the corresponding electronic case report forms. Any questions or suspicions arising during the process of checking the source and case report data shall be added to a formal list of queries, which will be addressed by the investigator filling out the data. If a problem is found, it will be processed and recorded in a timely manner. All documentation on quality control will be maintained to objectively assess the safety and key outcomes. The quality control personnel of the Shanghai Yueyang Integrated Hospital (Shanghai, China) will regularly monitor the data collected during the entire study period. The data monitoring committee will assess the safety data and critical efficacy outcomes after the trial is completed. Jiangsu Famaisheng Medical Technology, which is independent of the sponsor and investigators, will perform data audits in the middle of the trial. Interim auditing will include off-site surveillance and submission of a report on the progress of the study. All possible efforts will be made to maintain confidentiality of patient data by using multiple means, such as de-identification, use of password-protected secure servers, and restriction of access to study team members.

## Discussion

GA is caused by the deposition of MSU crystals in soft tissue, triggering severe but self-limited bouts of acute arthritis accompanied by intense pain, as well as articular and periarticular inflammation [19]. The acute attack of gout not only causes joint pain and dyskinesia, but more importantly, gout is associated with a number of complications, including hypertension, chronic kidney disease, cardiovascular disease, obesity, insulin resistance, diabetes, and hyperlipidemia [20–22].

In the early stages, gout often manifests as intermittent acute arthritis. A single joint is mainly involved, most commonly the first metatarsophalangeal joint. Joint swelling and pain usually last for 7 days, can be spontaneous or relieved by drugs, and do not have any symptoms during the interval [22]. With the extension of the course of gout, the number of attacks and the number of joints involved gradually increase, and joint symptoms begin to appear during the interval [23]. At the same time, the tophus is formed in the joint, skin, and soft tissue, and the joint is damaged or even maimed. Although gout treatment is effective, the compliance of patients with gout is unsatisfactory [24]. Poor compliance not only directly affects the therapeutic effect on patients but also places a heavy burden on the entire medical and health department [25–28].

TCM has been used for the treatment of gout for a long time, and its curative effect is remarkable. TCM considers that GA is caused by internal and external causes; the external causes mainly include wind, cold, dampness, and heat, while the internal causes mainly include the deficiency of vital *qi* of the human body, disorder of ascending clear and descending turbid function of spleen and kidney, and deficiency of *qi* and blood, leading to dampness-heat, turbid phlegm, stagnation of blood stasis, spleen deficiency, and other syndromes [29, 30], among which dampness-heat syndrome is the most common cause. Based on clinical practice, combined with the TCM-based pathogenesis of GA, our research group has formulated the TCM prescription of HZG, which is used to treat GA on the basis of the treatment principles of clearing heat, removing dampness, and dredging collaterals.

Previous research indicated that HZG has an anti-inflammatory effect on the downregulation of NALP3 and caspase-1 at the protein translation level [31]. Based on these previous observations, we cultured fibroblast-like synoviocytes stimulated by MSU with serum containing HZG and found that HZG inhibited the expression of IL-1β, TNF-α, and IL-6 [14]; this supports the therapeutic effect of HZG in patients with GA showing dampness-heat syndrome and motivated us to initiate this trial.

In this trial, we will use the granule formulation to minimize deviations associated with the use of herbs from different geographical regions, different varieties, and maintained under different storage conditions. We chose etoricoxib as a comparative drug. Etoricoxib is a new cyclooxygenase-2 (COX-2) inhibitor that can be used in the treatment of GA. The 2012 American College of Rheumatology guidelines for the management of gout indicate that etoricoxib has an evidence-based grade of A in the treatment of GA when using COX-2 inhibitors [32]. In this study, we aimed to clarify the efficacy of HZG treatment twice daily for 5 days. As acute GA is prone to recurrence, we emphasize a 1-week follow-up after the end of treatment.

We will carefully monitor the recurrence after the end of GA treatment. In addition, we will record all AEs and information regarding concomitant medications at each visit. The study results will help clarify the efficacy of HZG, as well as its safety in the treatment of GA in terms of both inflammation regression and rate of recurrence. We expect that this study will provide high-quality evidence that can be used to develop clinical treatment guidelines. Therefore, the results of this study are expected to have an important impact on public health.

The limitations of the study should be mentioned. Only one trial drug regimen will be tested, and the prescription will not be adjusted according to symptoms, which means that the results may be specific to the test regimen. In addition, because of the reliance on etoricoxib as the control drug in our study, our findings may not apply to the adverse reactions of other NSAIDs. Nevertheless, the findings will still be useful as a reference in clinical practice and will help pave the way for future research. Finally, the HZG prescription to be used in this trial was designed for the treatment of GA with dampness-heat syndrome; thus, the findings may not be applicable to other syndromes.

### Ethics and dissemination

Ethical approval was obtained from the Ethics Committee of Yueyang Hospital of Integrated Traditional Chinese and Western Medicine (approval no. 2020–024). Written informed consent will be obtained from all participants. We have uploaded the model consent form as supplementary material. No clinical data or bio-samples collected without participant consent. Informed consent forms for participation in a clinical trial and sample biobanking will be provided separately. The trial will be conducted in accordance with national laws, Good Clinical Practice guidelines, and the Declaration of Helsinki (2013).

We plan to publish the study results in scientific journals. The datasets used or analyzed during the current study will be available upon reasonable request from the corresponding author. We do not intend to separately inform participants about the results. Those individuals who have substantially contributed to the study design, implementation, and interpretation and reporting of clinical experimental protocols will qualify as authors of the final trial report. Reporting the study results is the responsibility of the clinical trial team.

### Trial status

The recruitment phase began on July 1, 2020. To date, 145 patients have been recruited. The estimated end date for this study is April 2023.

### Modification of the protocol

Any changes to the study protocol will be agreed upon by the project leader and the supervisor. The project team members and ethics committee will be notified before changes are implemented.
